# Sexual anxiety mediate relationship between sexual schemas and body image with sexual function in Iranian women

**DOI:** 10.1371/journal.pone.0305340

**Published:** 2024-07-26

**Authors:** Majid Yousefi Afrashteh, Rojan Blouri, Zekrollah Morovati

**Affiliations:** Department of Psychology, Faculty of Humanities, University of Zanjan, Zanjan, Iran; Padua University: Universita degli Studi di Padova, ITALY

## Abstract

**Background:**

Sexual function is one of the most critical challenges in human society, especially among women. The problems associated with sexual function are significantly ambiguous in a society like Iran. This study investigated the mediating role of sexual anxiety in relationship between sexual schemas and body image with female sexual function.

**Method:**

The research method was correlational and specifically path analysis. The statistical population included women aged 25–45 in Rasht, Iran, 2021. 365 women were selected using Cochran’s formula and the convenience sampling method. The Female Sexual Function Index (FSFI), Sexual Self-Schema Scale for Women (SSSS), Body Image Scale (BIS), and Multidimensional Sexual Self-Concept Questionnaire (MSQ) were used for data collection. For data analysis, SPSS-26 and LISREL 10.2 software were used.

**Results:**

Descriptive data analysis showed that mean and standard deviation were for further passionate-romantic schema 22.96 and 4.83, open-direct schema 27.64 and 5.09, embarrassed-conservative 20.93 and 4.61, body image 128.96 and 27.35, sexual anxiety 13.13 and 3.91 and sexual function 49.83 and 8.67. According to the results of path analysis, passionate-romantic (β = 0.51), explicit-comfortable (β = 0.27), shy-conservative (β = -0.59), and body image (β = -0.62) schemas showed a significant relationship with sexual function. Sexual function anxiety as a mediating variable also had a significant role (β = -0.41) in female sexual function.

**Conclusion:**

Sexual function anxiety, negative body image, and negative sexual schema negatively affect women’s sexual function, and positive sexual schemas and body image positively affect sexual function anxiety.

## Introduction

Today, sexual dysfunctions have become one of the most common phenomena in human society [1, 2]. Several factors are involved in problems related to family and marital relationships. Dissatisfaction with sexual relations between spouses is a critical factor [1]. According to the Fifth Edition of Diagnostic and Statistical Manual of Mental Disorders (DSM-5), there are different types of female sexual dysfunction, one of which is female interest/arousal disorder. female interest/arousal disorder is defined as Lack of interest or significantly reduced interest in sexual activity. At least three of the following symptoms occur for a minimum of six months: reduced interest in sex, reduced sexual thought or fantasies, reduced initiation of sexual activity, reduced excitement during sexual activity, reduced excitement to sexual cues, and reduced sensation during sexual encounters [1–3]. The prevalence of sexual dysfunctions in women worldwide is above 40% [4], while it is about 77% in Iran [5]. According to the DSM-5, sexual dynsfunctions “are a heterogeneous group of disorders that are typically characterized by a clinically significant disturbance in a person’s ability to respond sexually or to experience sexual pleasure. […] if the sexual difficulties are the result of inadequate sexual stimulation […] there may still be a need for care, but a diagnosis of a sexual dysfunction would not be made. These cases may include, but are not limited to, conditions in which lack of knowledge about effective stimulation prevents the experience of arousal or orgasm.” [6]. In Iran, reports indicate that 22% of young women are affected by sexual dysfunction, increasing by 75.7% in women over 40 [7].

Sexual self-schemas are some of the essential and effective personal factors in couples’ relationships and sexual satisfaction [8]. Anderson and Cyranowski see sexual schemas as the fundamental beliefs of a person that stem from past experiences and influence subjects. They do that by influencing the processing of sexual information on sexual experiences and behaviors. They categorize sexual schemas into positive sex schemas, which are passionate-romantic and explicit-open schemas, and negative sex schemas, which include shy-cautious schemas [9]. Respondents with positive schemas evaluate sexual behaviors positively and are more inclined to have sex [10]. Conversely, respondents with a negative view of sexual schemas are behaviorally restrained in their sexual and romantic relationships and conservative in their attitudes toward sexual dysfunctions [10–12].

Body image is defined as the mental image of a person’s body, attitudes about their body, appearance, health, normal functioning, and gender [13–15]. Positive body image is multifaceted and includes appreciation, acceptance, and love for the body [16, 17]. However, A negative image of the body leads to dissatisfaction with the appearance and causes a person to have negative beliefs about herself and avoid sensual experience in sex [18, 19].

Sexual performance anxiety is one of the consequences of negative body image. This is one of the most common sexual problems. Compared to other sexual problems, there is little agreed-upon specific solution for sexual anxiety [20]. Stress and anxiety for women in sex can have consequences including sexual dysfunction. Many different worries can lead to this problem: negative body image, weight, premature ejaculation, prolonged orgasm, and mood [21].

It has been reported that the regression analysis results show a significant relationship between sexual function and two sexual schemes: passionate-romantic and embarrassed-conservative [22]. In a study on Iranian married female students, results showed a significant positive relationship between marital satisfaction and the passionate-romantic and open-direct sexual schemes [23]. In another study on the relationship between Sexual Schemas and Sexual Satisfaction of Couples, results showed a significant positive relationship between sexual schemas and marital satisfaction [8]. In a study on stress, anxiety, depression and sexual dysfunction among postmenopausal women in Shiraz, Iran, results showed there was a significant relationship between stress, anxiety and sexual dysfunction [24]. Also, the relationship between body image and sexual function was examined in a relevant study. The results indicated that women’s higher body appreciation was associated with higher sexual satisfaction [18]. Bruce and Barlow’s research on investigating the nature and the role of functional anxiety in sexual dysfunction supported the relationship between sensory anxiety and sexual dysfunction [25]. In a study by McCabe on the role of functional anxiety in creating and maintaining sexual dysfunction in men and women, results showed the leading role of sexual anxiety in sexual dysfunction in men and women [26].

Although there are no specific theoretical models for women’s sexual performance in relation to sexual schemas and body image in the literature [27], it is possible to borrow from cognitive theories that propose self-representations and cause certain emotions and behaviors [28] In general, schemas are defined as filters through which people perceive, organize, and understand information about themselves and the world [29, 30] Schemas play an important role in human emotions and behaviors. Empirical studies on mood and anxiety disorders have shown that schemas play a key role in the development and maintenance of psychiatric disorders [31, 32] Also, theoretical foundations and research findings have provided convincing evidence for the clinical relationship between sexual schemas and sexual dysfunction [33]. In particular, models of women’s sexual functioning support the central role of schemas in sexual dysfunction [33, 34].

The relationship between sexual self-schemas and sexual performance with the mediation of sexual anxiety can be better understood by relying on the cognitive-behavioral model of sexual response. This model was first proposed by Barlow [35]. In this model, the main aspect of sexual performance problems is hypothesized to be anticipatory anxiety, which is the result of negative expectations. In such an anxiety-provoking situation, physiological arousal increases in the individual, which limits attention to sex-related cues. It is this diversion of attention from sex-related cues that reduces the sexual stimulation necessary to activate sexual arousal and performance. Finally, the vicious cycle—negative expectations—anticipatory anxiety—distraction from sexual cues—causes problems in sexual performance and affects sexual satisfaction. In such circumstances the individual’s own sexual schemas may be modified in such a way as to stabilize the sexual dysfunction, as it is now understood as a fundamental characteristic of the self. Consequently, women with damaged sexual schemas may be at risk of experiencing negative expectancies in sexual experiences that activate negative emotions such as anxiety before sexual activity and distract women from processing sex-related cues that are required for sexual arousal [27].

Researchers have found that a person’s sexual schema can influence cognitive processing and is an influential factor in how they respond to sexual information [36–38]. Compared to women with negative sexual schemas, women with positive sexual schemas experience better emotions towards sexual experiences and ultimately sexual activity. This positive emotion, by reducing anxiety, ultimately leads them to have more satisfying sex [39]. In Iran, the research of Tabatabai, Mousavi and Akbari [40] showed that negative sexual schemas increase sexual anxiety in men. In another study in Iran, it was found that sexual schemas both directly and indirectly, through increasing sexual anxiety, reduce the sexual performance of postmenopausal women [41]. Various studies have explained the relationship between body image and sexual performance [42–45]. In Iran, various studies have shown that interventions based on improving body image can be used to improve sexual function [46, 47]. According to Barlow’s model [35] in explaining sexual performance, in the present study, sexual anxiety was entered into the model as a mediator in the relationship between sexual schemas and sexual performance. In Barlow’s model, anxiety plays a mediating role between cognition and sexual performance. According to what has been reviewed, the research hypotheses are set as follows.

### Research hypotheses

- Passionate-Romantic sexual schema has a direct and positive relationship with sexual function.
- Open-Direct sexual schema has a direct and positive relationship with sexual function.
- Embarrassed-Conservative sexual schema has a direct and negative relationship with sexual function.
- Body Image have a direct and positive relationship with sexual function.
- Sexual anxiety has a direct relationship with sexual function.
- Passionate-Romantic sexual schema has a direct and negative relationship with sexual anxiety.
- Open-Direct sexual schema has a direct and negative relationship with sexual anxiety.
- Embarrassed-Conservative sexual schema has a direct and positive relationship with sexual anxiety.
- Body Image have a direct and negative relationship with sexual anxiety.
- Passionate-Romantic sexual schema has an indirect relationship (Through the mediation of sexual anxiety) with sexual function.
- Open-Direct sexual schema has an indirect relationship (Through the mediation of sexual anxiety) with sexual function.
- Embarrassed-Conservative sexual schema has an indirect relationship (relationship (Through the mediation of sexual anxiety) with sexual function.
- Body Image have a direct relationship with sexual function.

## Research methods

### Participants

The statistical population included women aged 25–40 living in Rasht, Iran, in 2021. Participants were 365 women selected by the convenience sampling method and according to Cochran’s sample size formula. The inclusion criteria included marriage, being in the 25–45 age range, having no menopause, acute physical and mental problems, no use of psychiatric drugs, no experience of mourning in the last two months before the survey, and giving informed consent to participate. The exclusion criteria included non-response to more than 15% of the questionnaire items and loss of appropriate cooperation conditions, such as illness. Of 365 participants, 50% were in the 25–30, 30% in the 31–35, and 20% in the 45–36 age group. Also, 56% were homemakers, and 44% had a university degree. More details are given in Table 1.

**Table 1:** Demographic statistics of the subjects.

variable	Frequency	Percent
**Age**		
25–30	162	44
31–35	98	27
36–40	69	19
41–45	36	10
Total	365	100
**Education**		
Diploma or lower education	205	56
Associate Degree	81	22
Bachelor	36	10
Masters	28	7.5
Ph.D.	15	4.5
**Job**		
housewives	204	56
Employed	161	44

### Procedure

**Fig 1:**
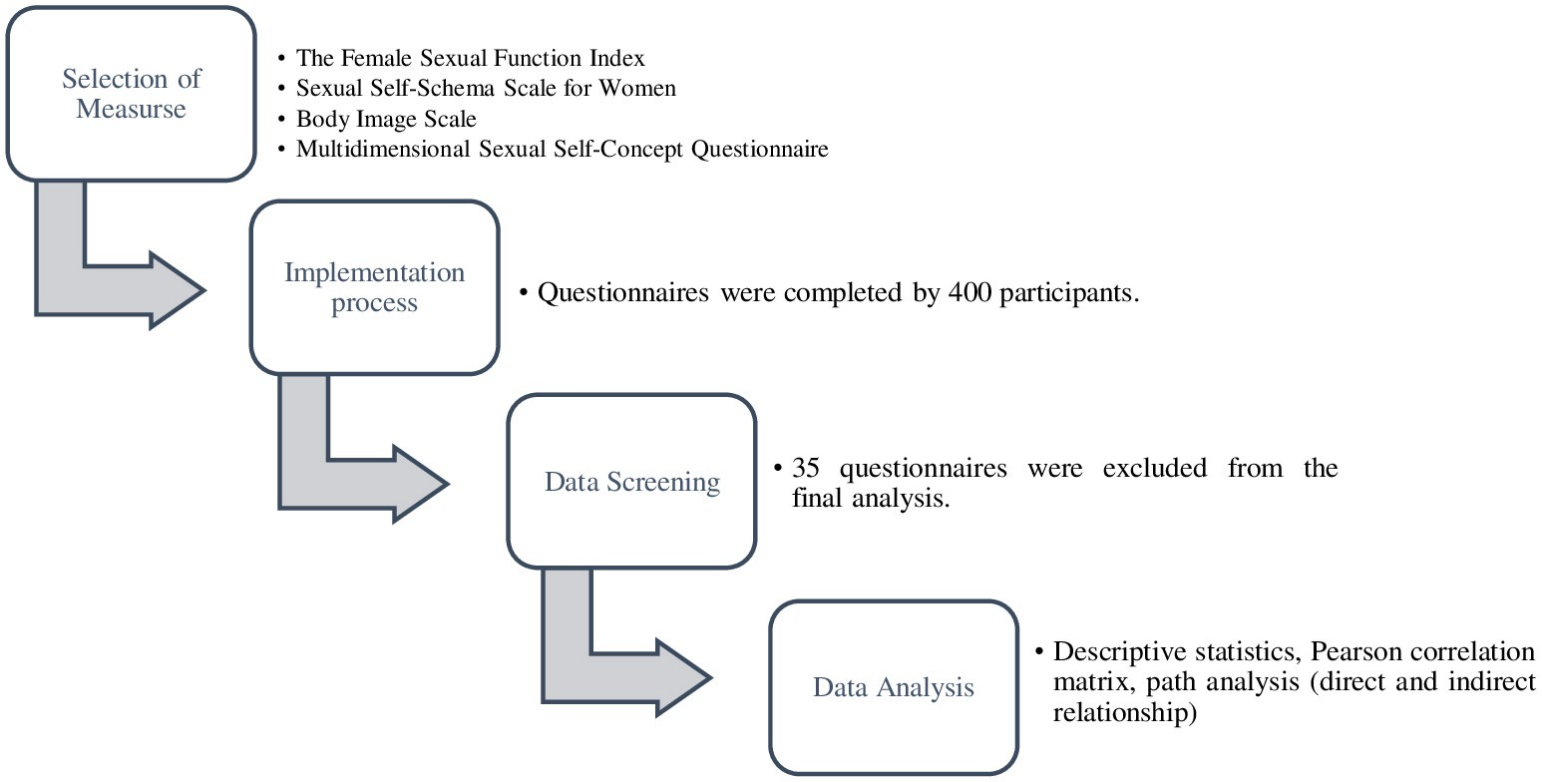
Flowchart of the research method.

This cross-sectional study was conducted from April 15 to June 8, 2021. After obtaining the necessary permits and letters of introduction, the study population was formed based on the entry criteria. Research instruments were prepared face-to-face and online. In order to collect data, the Central Health Center of Rasht was referred, and eligible women were asked to complete the questionnaires after reviewing the entry criteria. Informed consent was obtained from all participants and their legal guardians in accordance with the Declaration of Helsinki. In the face-to-face implementation, the objectives of the research were explained to the participants and they were asked to read the informed consent form and sign it if they agreed. In the online implementation, before viewing the questions of the questionnaires, the informed consent form included the objectives of the research, which needed to be approved by the participant. The questionnaires were initially distributed among 400. After excluding 35 participants (15 participants did not fill out the informed consent form correctly. 12 questionnaires had more than 15% missing. The data of 8 questionnaires were found to be outliers), the data of 365 women were included in the final analysis. The response rate was finally 0.91. All rights of the participants were protected during this study. The procedures performed in the study involving human participants were according to the ethical standards of the National Research Committee. This study was approved by department of psychology, University of Zanjan, Zanjan, Iran. Approval and monitoring of ethical cases was done in this department. Subjects completed a consent document before the survey and were allowed to leave the study at any time. The flowchart of the research method is shown in Fig 1.

### Instruments

#### The Female Sexual Function Index (FSFI)

This scale [48] includes 19 questions with five or six options ranging from *none* to *almost always* in the areas of female sexual function. In the desired areas, psychological arousal, vaginal lubrication, orgasm, satisfaction, and pain are measured. The minimum and maximum scores are 7 and 95. It should be noted that the test validity in Iran was assessed by Mohammadi et al. [49]. Cronbach’s alpha coefficient in all subjects for each domain and the whole scale was equal to or over 0.70, which is consistent with the results of research by Rosen et al. [48] (0.89) and Wiegel et al. [50] (0.80). Cronbach’s alpha of the Sexual Function Index in the present study was 0.89.

#### Sexual Self-Schema Scale for Women (SSSS)

This scale has 50 traits on a 7-point Likert scale (from 0 = very low to very high = 6) in which the extent each trait describes the respondent is determined. As people do not talk freely about their sexuality, 24 traits are used as fillers in this test to hide the central nature of the assessment from the subject. This scale has 26 main items and three subscales: passionate-romantic, open-direct, and embarrassed-conservative. Other items are fillers used to cover the central nature of the test and are not counted in the scoring. This scale was developed in 1994 by Anderson and Cyranowski. The minimum and maximum scores that can be obtained on this scale are 0 and 300. In Anderson and Cyranowski’s research, Cronbach’s alpha coefficient was 0.81 in the passionate-romantic subscale, 0.77 in the open-direct subscale, 0.66 in the embarrassed-conservative subscale, and 0.82 in the total subscale, which is desirable [51]. In Iran, Mojtabaei et al. determined the scale reliability to be 0.78. Also, the reliability of the whole scale and subscales has been calculated above 0.7 [22]. In present study Cronbach’s alpha coefficient was 0.84 for passionate-romantic subscale, 0.77 for open-direct subscale and 0.80 for embarrassed-conservative subscale.

#### Body Image Scale (BIS)

This scale contains 46 items that are responded on a 5-point scale from 1 to 5 (very dissatisfied = 1, dissatisfied = 2, moderate = 3, satisfied = 4, very satisfied = 5) [52]. The minimum and maximum scores obtained in this test are 46 and 230. Areas to be measured on this scale include head and face, upper limbs, lower limbs, and general attitudes toward body characteristics. In Iran, the test validity was reviewed and approved by Yazdanjoo in 2000 on 99 high school students. The correlation coefficient calculated by the retest test was 0.84 [53]. Mojtabaei et al. Examined BIS on a sample of 190 nurses. Cronbach’s alpha coefficient for the facial subscale was 0.82, in the upper limbs was 0.78, in the general attitude subscale was 0.81, and the overall score [22]. Cronbach’s body image scale in the present study was 0.90.

#### Multidimensional Sexual Self-Concept Questionnaire (MSQ)

The Sexual Self-Concept Questionnaire has been used in many studies and has 12 subscales, one of which is sexual anxiety. It consists of 5 items. In this questionnaire, the subject on a 5-point Likert scale indicates the degree of agreement or disagreement with each statement. This scale was compiled by Snell [54] and translated into Persian by Rajaei et al [55]. Each item is assigned a score between 0 and 4. The minimum and maximum scores obtained in this questionnaire are 0 and 20, respectively. A high score on the Sexual Anxiety Questionnaire indicates a high level of sexual anxiety. In a study by Snell et al., Cronbach’s alpha coefficient was 0.83 [54]. In research by Rajaei et al., Cronbach’s alpha coefficient was determined to be 0.89 [55]. Cronbach’s alpha, the sexual function anxiety index scale, was 0.86 in the present study.

### Statistical analysis

SPSS v.26 (IBM) and LISREL v10.2 were used for data analysis. Descriptive analyses, including mean and standard deviation and Pearson correlation matrix with SPSS and path analysis, were performed using LISREL. Although in large samples, normality is less critical [56], in this study, the indices of Skewness and kurtosis were examined. As shown in Table 2, these indices are between -1 and 1 for all variables, so the data distribution is normal, and there is no problem with using Pearson correlation and path analysis. Path analysis with ordinal data was conducted using the diagonally weighted least squares method (WLSMV). The model fit indices were Chi-square statistics, Chi-square/df, Root Mean Square Error of Approximation (RMSEA), Comparative Fit Index (CFI), Tucker-Lewis Index [TLI, also known as the non-normed fit index (NNFI)], Goodness of Fit Index (AGFI) and Adjusted Goodness of Fit Index (AGFI). The model was judged as having a good fit when the overall picture of fit indices indicated good fit and excellent if all of them indicated well fit: RMSEA ≤ 0.05, CFI and TLI ≥ 0.95, and WRMR < 0.90 [57]. Likewise, a significant PCLOSE (p<0.05) indicates that RMSEA > 0.05 (and therefore, it is not a good model).

**Table 2:** Descriptive statistics for the research variables.

Variable	Dimensions	M	SD	SK	KUR
Sexual Schema	Passionate-Romantic	22.96	4.83	-0.30	-0.88
	Open-Direct	27.64	5.09	-0.91	0.79
	Embarrassed-onservative	20.93	4.61	-0.02	-1.99
Body Image		128.96	27.35	-0.21	-0.38
Anxiety Sexual		13.13	3.91	0.27	-0.83
Sexual Function		49.83	8.67	-0.40	-0.75

## Results

Descriptive statistics including mean, standard deviation, Skewness (SK) and kurtosis (KUR) are reported in Table 2.

Table 2 shows the descriptive information including mean, standard deviation and skew index for research variables. The skewness index for all variables is between -1 to +1 and therefore their normality is confirmed. The matrix of Pearson correlation coefficients for the relationship between the variables is reported in Table 3.

**Table 3:** Correlation matrix between research variables.

**Variables**	**1**	**2**	**3**	**4**	**5**
1-Passionate-Romantic	1				
2-Open-Direct	0.23**	1			
3-Embarrassed-Conservative	-0.34**	-0.43**	1		
4-Body Image	0.08	0.19**	-0.15**	1	
5-Sexual Anxiety	-0.44**	-0.46**	0.48**	-0.23**	1
6-Sexual Function	0.46**	0.41**	-0.45**	0.25**	-0.50**

According to the results of Table 3, the correlation coefficient of Sexual Function with the Passionate-Romantic schema is 0.46, with the Open-Direct schema 0.41, with the Embarrassed-conservative schema is -0.45 and with the body image is 0.25. Also, the correlation coefficient of sexual anxiety with sexual function is -0.50.

Also, the correlation coefficient between age, education level and job status with sexual function was -0.32 (p<0.01), -0.06 (p = 0.28) and 0.03 (p = 0.57), respectively. Due to the significant relationship between age and sexual function, age was entered into the model and analyzed as a control variable. However, due to the non-significance of the relationship between education level and job status with sexual function, they were not included in the analysis model.

**Fig 2:**
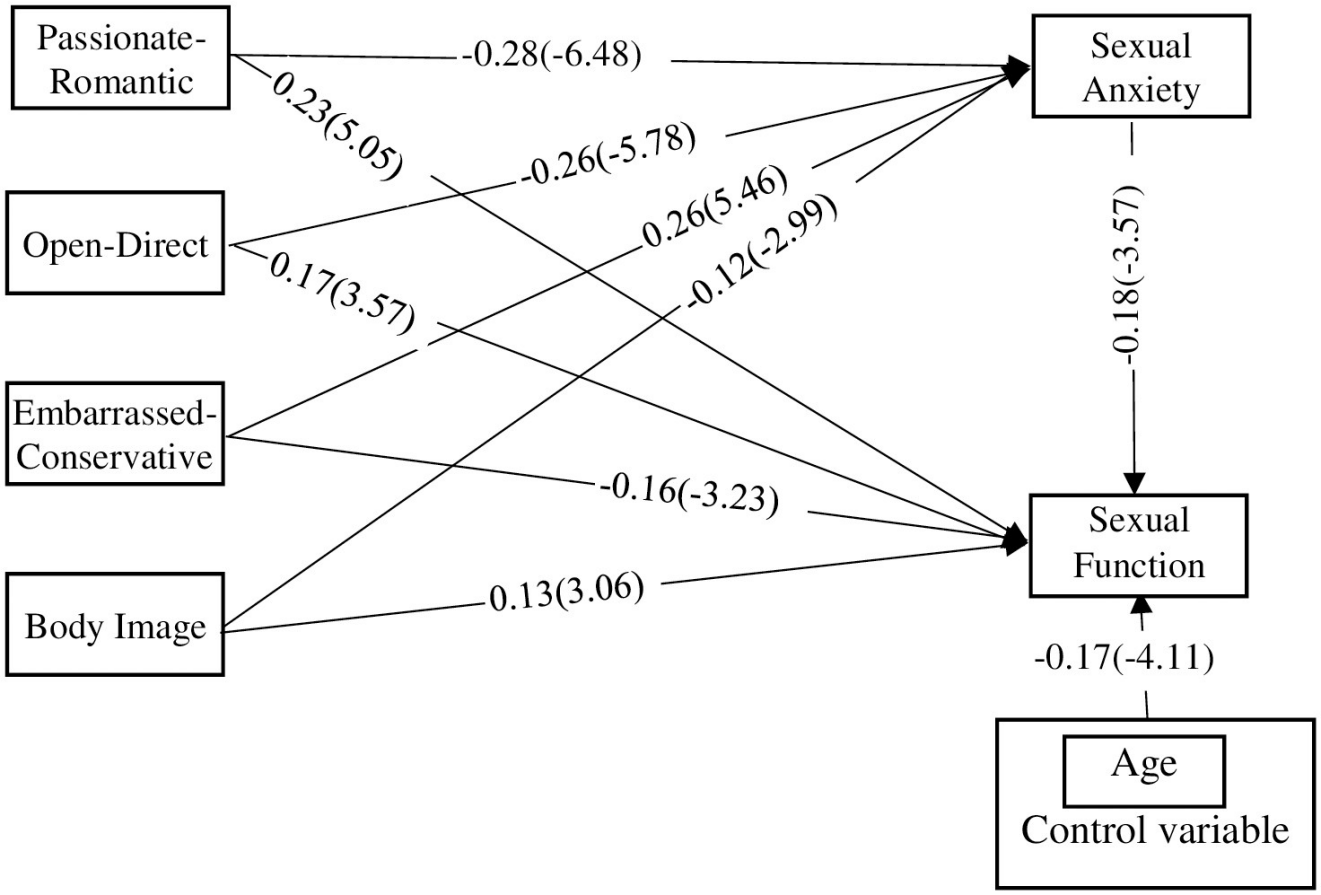
Standard path coefficients and t values (in parentheses).

According to the results of path analysis shown in Table 4, Passionate-Romantic schema (β = 0.23), Open-Direct schema (β = 0.17), Embarrassed-Conservative (β = -0.16) and body image (β = 0.13) have a significant direct relationship with sexual function. Passionate-Romantic schema (β = -0.28), Open-Direct schema (β = -0.26), Embarrassed-Conservative (β = 0.26) and body image (β = -0.12) have a significant relationship with sexual anxiety. Also, Finally, the pathway of sexual anxiety is significant with sexual function (β = -0.18). Furthermore Passionate-Romantic schema (β = 0.052), Open-Direct schema (β = 0.049), Embarrassed-Conservative (β = -0.047) and body image (β = 0.023) have a significant indirect relationship with sexual function. Therefore, the research hypotheses indicating a direct relationship between sexual schemas and body image with sexual function. Also the mediating role of sexual function anxiety in this correlation were confirmed. Fig 2 shows the standard parameter and the t-value for each of the paths.

**Table 4:** Path coefficients for direct, indirect and total effect of sexual schemas and body image on sexual function.

Path	Standard estimate	t-value	P-value
**Direct effect**			
Passionate-Romantic **to** Sexual Function	0.23	5.05	P<0.001
Open-Direct **to** Sexual Function	0.17	3.57	P<0.001
Embarrassed-Conservative **to** Sexual Function	-0.16	-3.23	P<0.01
Body Image **to** Sexual Function	0.13	3.06	P<0.001
Sexual Anxiety **to** Sexual Function	-0.18	-3.57	P<0.001
Age **to** Sexual Function	-0.17	-4.11	P<0.001
Passionate-Romantic **to** Sexual Anxiety	-0.28	-6.48	P<0.001
Open-Direct **to** Sexual Anxiety	-0.26	-5.78	P<0.001
Embarrassed-Conservative **to** Sexual Anxiety	0.26	5.46	P<0.001
Body Image **to** Sexual Anxiety	-0.12	-2.99	P<0.01
**Indirect effect**			
Pa-Ro **to** SF **through** SA	0.052	3.13	P<0.01
O-D **to** SF **through** SA	0.049	3.04	P<0.01
Em-Co **to** SF **through** SA	-0.047	-2.99	P<0.01
BI **to** SF **through** SA	0.023	2.29	P<0.05
**Total effect**			
Passionate-Romantic **to** Sexual Function	0.28	6.41	P<0.001
Open-Direct **to** Sexual Function	0.22	4.73	P<0.001
Embarrassed-Conservative **to** Sexual Function	-0.20	-4.30	P<0.001
Body Image **to** Sexual Function	0.15	3.59	P<0.001

Pa-Ro = Passionate-Romantic; O-D = Open-Direct; Em-Co = Embarrassed-Conservative; BI = Body Image; SA = Sexual Anxiety

Goodness-fit indices are reported in Table 5. According to the results of this table, the final model has a good fit.

**Table 5:** Indices of goodness of fit.

Index	χ2/df	RMSEA	GFI	AGFI	NFI	NNFI	IFI
Acceptable values	<3	<0.08	>0.9	>0.9	>0.9	>0.9	>0.9
Observed values	2.45	0.06	0.99	0.95	0.99	0.94	0.99

## Discussion

This study aimed to determine the relationship between sexual schemas and body image with female sexual function by analyzing the mediating role of sexual function anxiety. The results supported the research hypotheses. So that the two sexual schemes of Passionate-Romantic and Open-Direct had a negative relationship with sexual function anxiety and a positive relationship with sexual function. Also, Embarrassed-conservative schema and body image were positively related to sexual anxiety and negatively related to sexual function. The adverse effect of sexual function anxiety as a mediator on women’s sexual function was also confirmed.

Confirmation of the relationship between sexual schemas and sexual function is consistent with various studies. In a study by Mojtabaei et al. on Iranian women, the regression analysis results showed a significant relationship between two sexual schemes of Passionate-Romantic and Embarrassed-conservative with sexual function [22]. Zargarinejad and Ahmadi’s study supported the mediating role of sexual schemas in sexual function and sexual satisfaction in Iranian married women [58]. In a study by Maryami et al. on Iranian married female students, the results showed a significant positive relationship between marital satisfaction and two sexual schemas: Passionate-Romantic and Open-Direct [23]. Of course, the relationship between sexual schemas and sexual function has been studied and confirmed in other studies [22, 59, 60]. Explaining this finding, we can say that people’s cognitive structures are formed and transformed through interaction with the environment. These mental structures that evolve from childhood to adulthood eventually spread to all aspects of a person’s life. In an interpersonal relationship, the mental structures of the two parties may overlap. On the other hand, couples create positive and negative attribution [61]. Such attributions can affect the feeling and quality of the couple’s relationship. These attributions are due to mental structures and underlying schemas [62]. The role of schema in creating expectations of the other party’s emotional behavior is another explanation. Schemas deviate from the formation of expectations and predictions of the emotional partner, and expectations shape a person’s behavior [63].

The relationship between body image and sexual function has also been confirmed in various studies [64–66]. Nazarpour et al. found a significant positive relationship between body image and all components of sexual function in postmenopausal Iranian women [64]. In a study by Winter et al., the results showed that higher body appreciation was associated with higher sexual satisfaction in women [18]. Some studies have also shown that lower body image is associated with high levels of orgasmic disorders [65]. Positive body image and high acceptance of physical appearance are associated with good orgasm and sexual function [66]. This finding can be explained by the notion that women’s focus on their bodies may divert their attention from their partner’s positive sexual feelings and sexual behaviors. This leads to decreased sexual self-efficacy and sexual function. Thus, a negative body image may reduce sexual desire, intimacy with a partner, sexual response, and proper sexual function [65].

This study confirmed the role of sexual function anxiety on female sexual function. This result is consistent with the results of various studies [24–26]. For example, Bruce and Barlow’s findings supported the relationship between sensory anxiety and sexual dysfunction [25]. In a study by McCabe to investigate the role of functional anxiety in creating and maintaining sexual dysfunction in men and women, the results showed the leading role of sexual anxiety in sexual dysfunction in men and women [26]. The research by Yazdanpanahi et al. supported the negative role of stress, anxiety, and depression in women’s sexual function in Iran [24]. In the present study, sexual function anxiety was investigated as a mediator. So that it is affected by underlying schemas and affects sexual behavior, this role was less considered in previous research. Anxiety during sexual intercourse leads to physiological arousal in the person and thus facilitates attention to unrelated sexual signs. This shift in attention to sexually unrelated signs reduces the sexual arousal needed for effective sexual function. Eventually, the vicious cycle begins with negative expectations, leading to sexual anxiety, a distraction from sexual signs, and sexual dysfunction. Sexual satisfaction is the last link in this chain [66].

There are some methodological limitations in this research that should be considered. The first limitation of this research relates to not dividing the model analysis results based on subscales of sexual function. This was done in accordance with the objectives of the study, but more details may be provided by analyzing the subscales of sexual function. Therefore, the results of this study are limited to the total score of sexual function and cannot be generalized to all its subscales. Future researchers are recommended to analyze the subscales and compare the results. Second, the present study is of self-report, which may be affected by social desirability limitation inherent in most research [67], and there should be caution in citing the research results. Future research can minimize this problem by taking precautions to combat socially desirable responses recommended by Mick [68]. The third limitation of this research is related to its cross-sectional implementation, which limits the application of causal conclusions [69]. Researchers are recommended to re-examine causal relationships through experimental or longitudinal methods.

### Clinical implications

Sexual dysfunction in Iranian women is a taboo problem, and few women seek treatment. Similarly, there are few research findings in this regard. This study identified the role of sexual schemas and body image in sexual dysfunction. However, the new finding of this study was the mediating role of sexual anxiety. Indeed, it was found that sexual anxiety is the mechanism of the effect of sexual schemas and body image on sexual dysfunction. Cultural factors largely influence sexual schemas, and preventive intervention programs are needed to improve them. However, schema therapy can improve these schemas. Sexual anxiety is more curable than schemas and can be the most appropriate goal of sex therapy. Psychotherapists are advised to focus on improving women’s sexual schemas, body image, and sexual anxiety to improve women’s sexual function.

## Conclusion

Sexual performance in women, especially in Iran, is a closed and neglected issue. Many women in families refuse to express their sexual dysfunctions, and their treatment is minor. This study and similar studies can help break the taboo on women’s sexual dysfunctions. The results of this study supported the confirmation of the research hypotheses. It was found that sexual schemas are present as underlying factors in women’s sexual anxiety. These schemas are formed throughout life and influenced by the environment and informal learners. To modify these schemas, attitudes toward emotional relationships can be improved in women in need. Schema therapies can be helpful for this purpose. The results showed that anxiety plays a mediating role between schemas and sexual function. If the level of sexual function anxiety in a person is high, treatment of anxiety alone can temporarily improve sexual function. Continuing research in this area can be promising and guide research that strengthens marital relationships. This research also had some limitations: In this study, only a questionnaire was used, which may affect the results by biasing the subjects’ answers. This research was correlational because it makes it challenging to conclude causality. Also, it was difficult to determine and control the psychological problems of participants. Another limitation of this study was the lack of control throughout the marriage and marital satisfaction. In future research, it is suggested that researchers consider the type of marriage, marital satisfaction, and psychological status and use qualitative interviews.
